# Quantifying the size and characteristics of a population of people who use drugs on the reservation lands of a tribal nation in the southern plains (USA)

**DOI:** 10.1186/s12954-025-01194-z

**Published:** 2025-04-04

**Authors:** Sean T. Allen, Molly C. Reid, Kristin E. Schneider, Allison O’Rourke, Brady A. Garrett, Maisie Conrad, Coleman Cox, Kendra Lewis, Sierra Lewis, Lisa Wilson, Melissa Walls

**Affiliations:** 1 Department of Health, Behavior and Society, Johns Hopkins Bloomberg School of Public Health, 624 N. Broadway, Baltimore, MD 21205 USA; 2 DC Center for AIDS Research, Department of Psychological and Brain Sciences, George Washington University, 20052 Washington, DC USA; 3 Cherokee Nation Public Health, 1325 E. Boone, Tahlequah, OK 74464 USA; 4 Department of International Health, Johns Hopkins Bloomberg School of Public Health, 624 N. Broadway, Baltimore, MD 21205 USA; 5 Cherokee Nation Harm Reduction Program, 214 N. Bliss Ave, Tahlequah, OK 74464 USA; 6 Cherokee Nation Infectious Disease, 19600 E Ross St, Tahlequah, OK 74464 USA; 7 Peaceful Way, 1011 S. Muskogee, Tahlequah, OK 74464 USA

**Keywords:** People who use drugs, Population Estimation, Addiction, Overdose, Indigenous peoples

## Abstract

**Background:**

Reversing trends in substance use-related health inequities among Indigenous Peoples requires investments in epidemiological research anchored in community-based participatory research (CBPR) methodologies. There is scarce literature that describes how to implement population estimation methods on American Indian reservation lands.

**Objective:**

This research describes how we leveraged CBPR throughout the implementation of a population estimation study conducted in collaboration with a Tribal Nation in the southern plains to quantify the size and characteristics of persons with histories of illicit substance use on reservation lands.

**Methods:**

We used the capture and recapture population estimation methodology in April-May 2023 to estimate the size of the population of people who used illicit substances in the past six months in a county within the collaborating tribe’s jurisdiction. Participant recruitment occurred in areas where people who use drugs were known to congregate. Participants completed a survey that included measures pertaining to sociodemographics, substance use, harm reduction, overdose, sexual health, and cultural factors.

**Results:**

In total, *N* = 501 surveys were completed by unique persons who had used illicit substances in their lifetime. A large proportion had injected drugs in the past six months or greater than six months ago (19.6% and 31.7%, respectively). There were *N* = 210 persons who reported having used illicit substances by at least one route of administration within the last six months. We estimated that there were 419 (95% confidence interval = 277, 562) adults who had recently used an illicit substance in the county where the study occurred.

**Conclusions:**

This study demonstrates that population estimation methodologies can be integrated with community-based participatory research approaches to quantify the size of populations of people who use drugs. Future work should be conducted to understand the degree to which population-level needs evolve over time and in response to local initiatives.

## Background

The addiction and overdose crisis disproportionately affects American Indian/Native American (hereafter, “Indigenous”) Peoples in the United States (US) [1–3]. From 2019 to 2020, overdose fatality rates increased in 25 states and the District of Columbia with American Indian/Alaska Native (AI/AN) persons experiencing a 39% relative rate increase [4]. In 2020, overdose mortality rates were highest among AI/AN persons (41.4/100,000), nearly 31% greater than White people [2]. The scale of the overdose crisis in the US is a contributing factor to recent declines in life expectancy among Indigenous Peoples, which are lower than all other racial and ethnic groups [5]. Injection drug use (IDU)-associated harms (e.g., bloodborne infections disease transmission) are concomitant public health crises among Indigenous Peoples [6–10]. For example, since 2004, Indigenous Peoples have had the highest rates of acute hepatitis C virus infection [6]. Centers for Disease Control and Prevention (CDC) data also indicate that, in 2020, Indigenous Peoples had the largest proportion (47%) of HIV diagnoses associated with IDU than any other racial or ethnic group [11]. To reverse these inequities, there is a sustained need for interventions that center Indigenous cultures and contexts and build on sources of strength and healing [12–14].

Health inequities, including those associated with substance use, among Indigenous Peoples are driven by a multiplicity of factors, including: structural racism, historical and intergenerational traumas, socioeconomic factors, cultural assimilation, and limited access to healthcare [15–17]. Notably, research documents that cultural practices and community engagement are protective against adverse health outcomes among Indigenous Peoples [18–20]. However, access to sources of strength and healing (e.g., ceremonial practices, community-based supports) may be obstructed by unjust policies and systems anchored in colonialism that afford few protections for engagement in traditional Indigenous approaches to well-being [21]. Further, in many instances, evidence-based response services (e.g., medications for opioid use disorder, cognitive behavioral therapy) are unavailable on reservation lands, or require Indigenous Peoples to travel long distances to access services that do not integrate traditional cultural practices and values [22].

Reversing trends in substance use-related health inequities among Indigenous Peoples requires sustained investments in epidemiological research. In part, this need is driven by the racial misclassification of Indigenous Peoples in much of the scientific literature [23–27]. For example, many studies do not adequately engage Indigenous Peoples in recruitment efforts, leading investigators to assign “Other Race” or indicate that there were so few Indigenous participants that their data are not viable analytically [28]. Racial misclassification of Indigenous Peoples is a direct contributor to a cycle of inadequate funding for the implementation of evidence-based response initiatives [29]. For instance, lacking timely and accurate data about the scale of substance use and associated inequities in Indigenous communities may diminish capacities to secure competitive grant funding. Not knowing the true scale of substance use inequities may also lead Indigenous communities to struggle to develop comprehensive response plans that maximize the public health utility of finite funding [29, 30]. Notably, ethical approaches to research with American Indian/Alaska Native Peoples requires attention to and support for Indigenous self-determination and Tribal sovereignty (i.e., Tribes are sovereign Nations that can regulate research on their own lands) [31, 32]. One commonly used orientation for promoting these tenets is community-based participatory research (CBPR), an approach that works to shift power structures such that communities and researchers are equal partners working collaboratively to address community prioritized issues [33, 34]. CBPR is designed to prioritize and integrate community knowledge and perspectives within research questions and methodologies, thereby enhancing the relevance and rigor of studies [32, 34].

Population estimation methodologies can be used to quantify the size and characteristics of populations (e.g., refugee populations, sex workers, people who use drugs) to inform resource allocation [35–43]. These methodologies have been applied throughout the world, resulting in a robust evidence-base for their public health utility [35–43]. In recent years, for example, population estimation studies were conducted in West Virginia and the District of Columbia to ascertain the number of people who inject drugs (PWID) [35, 36]. Data from population estimation studies can be used in nuanced examinations of factors associated with high-risk behaviors and health outcomes. For instance, data from a 2018 PWID population estimation study afforded examinations of factors associated with: high-risk injection practices, injection socialization behaviors, overdose, harm reduction services utilization, polysubstance use, interest in pre-exposure prophylaxis for HIV prevention, and engagement in substance use disorder treatment [44–52]. Given the combination of substance use-associated health inequities among Indigenous Peoples and the consequences of racial misclassification, population estimation methodologies may afford tribes enhanced capacity to respond to the addiction and overdose crisis via informing local understandings about the scale of population-level needs for services.

The scientific literature has many examples of applications of population estimation methodologies [35–42]. Unfortunately, none describe the process of conducting population estimation studies in collaboration with Indigenous Peoples and implementing them on American Indian reservation lands. This gap is likely reflective of a confluence of several factors, including inadequate funding for research initiatives led by tribes, the scientific community failing to involve Indigenous communities as equal partners in studies, and historical injustices that led to justifiable mistrust of “research” among Indigenous communities. Leveraging CBPR methodologies may provide an equity-driven approach for informing population estimation studies conducted among Indigenous Peoples. The purpose of this research is to describe how we conducted a population estimation study that was anchored in principles of CBPR to quantify the size and characteristics of persons with histories of illicit substance use in a county within the jurisdiction of a Tribal Nation in the southern plains (United States).

## Methods

### Study overview

We partnered with a Tribal Nation in the southern plains of the United States to implement this study. In alignment with principles of CBPR, members of a project supported Community Research Council (CRC) served as partners and co-leaders throughout this project. The CRC included members of the collaborating Tribal Nation with lived or professional experiences related to substance use and persons who provided healthcare services on reservation lands. Several CRC members also resided in the county where this study occurred. The CRC did much more than simply “advise.” For example, they worked with the academic investigators to identify areas of adaptation for the population estimation methodology. They also worked collaboratively to tailor the survey instrument such that it was locally relevant, actionable, and respectful of Indigenous culture and context. During data collection, the CRC supported participant recruitment via serving as trusted community members who disseminated information about the study to people who used drugs. Subsequently, the CRC reviewed all study findings and supported the interpretation of results.

### Study setting

In consultation with the CRC, one county within the jurisdiction of the collaborating Tribal Nation was selected as the focal area for this study. Existing data suggest the county is disproportionately affected by overdose morbidity and mortality. The majority of the focal area is considered rural by the US Census Bureau [53]. In July 2022, there were just over 30,000 residents of the focal county that were at least 18 years old. Approximately one quarter of residents of the focal county identify as “American Indian and Alaska Native alone” according to the US Census Bureau.

### Population estimation methodology

We used the capture and recapture population estimation methodology in our study. Direct applications of this methodology require primary data collection among the target population in which persons are counted across two periods of data collection (i.e., the capture and recapture phases) [43]. Persons who participated in both periods of data collection are considered “recaptures” and, in combination with the number of participants in each period, affords a population estimate [43]. The mathematical formulas for calculating a population estimate and associated 95% confidence interval are shown in Appendix A.

### Inclusion criteria

Inclusion criteria were discussed with the CRC and other community members to determine the requirements that would maximize participation without being intrusive. A consensus was reached that persons may be reticent to verbally disclose to study staff the recency of their substance use behaviors and/or what substances they used due to stigma. As a result, the eligibility criteria were broad: (1) to be at least 18 years old; and (2) to have ever used drugs by any route of administration. These criteria are similar to those used in other population estimation studies involving persons who use drugs [35, 36].

### Recruitment

Areas in the community for participant recruitment were primarily identified via discussions with the CRC and other constituents in the focal county who were familiar with the local population of people who use drugs. Through these discussions, we identified a list of potential recruitment locations, including public parks, hotels, neighborhoods, and agencies and organizations that served people who use drugs. Prior to launching data collection efforts, our team conducted windshield tours in which we drove to potential recruitment locations to identify safety concerns and engage local community members in discussions about the viability of recruitment at each venue. In many instances, leadership at the venues we visited were extremely supportive of our study and allowed our team to set up tables on site (e.g., in parking lots, conference rooms, waiting areas, public spaces) to support recruitment. When feasible, appropriate, and with the permission of venue owners, we also set up tents to provide shade for participants as they took the survey. Additionally, we grilled hot dogs and offered other snacks/drinks to community members regardless of their engagement in the study to bolster support.

### Capture & recapture data collection phases

The capture phase occurred in April 2023 and lasted 6 days. Two weeks later, the recapture phase launched in May 2023 and lasted four days. Study staff approached persons at recruitment sites and informed them about our study. In many instances, community members approached our study team as they were already familiar with our project via dissemination activities led by CRC members and other participants. Persons who expressed interest in participating were screened for eligibility by study staff. All participants provided verbal consent prior to completing the data collection procedures. During the capture and recapture phases, participants received a $20 Amazon gift card and a $20 Visa gift card, respectively, as an incentive. We selected these incentives as they were memorable and used to identify recaptures (i.e., persons who participate in both phases of data collection). The survey included items that asked if participants had previously completed the survey and received either of the incentives. These questions allowed us to exclude in-phase duplicates (i.e., persons who had taken the survey more than once during a single phase of data collection) and quantify the number of unique persons who participated in both study phases. All data were collected anonymously and via audio computer-assisted self-interview (ACASI) on tablets. All questions and answers were read to participants via audio recordings made by a local community member to reduce bias.

### Survey instrument

The survey instrument included a number of measures pertaining to sociodemographics, structural disadvantage, substance use, and harm reduction. Sociodemographic measures included questions that ascertained age, gender, sexual orientation, race, ethnicity, and relationship status. We also asked persons if they were an enrolled citizen of the collaborating Tribal Nation (i.e., they had a tribal citizenship card). Participants who self-identified as American Indian were asked a series of items that measured cultural identity [54]. Structural disadvantage measures included items related to highest level of educational attainment, employment, housing, health insurance coverage, recent arrest, engagement in transactional sex work, and hunger. Substance use measures included questions that ascertained the recency with which they injected, smoked, snorted, and ingested drugs to get high. Persons who reported recent (past 6 months) engagement in a modality of drug use were asked follow up questions about the substance(s) they used (e.g., heroin, methamphetamine). Participants were also asked about sexual health (e.g., recency of HIV/STI testing) and overdose experiences. Harm reduction measures included questions that assessed awareness of fentanyl test strips, interest in the use of public health vending machines and needle exchange programs if they were available, and willingness to distribute naloxone and HIV self-test kits.

### Analyses

Data were analyzed using SAS 9.3. We focused our analyses on persons who reported having recently (i.e., past six months) used illicit drugs. Pearson’s Chi square tests and independent samples t-tests were used to test for differences between persons who identified as American Indian and White. Sample size limitations did not afford comparisons between other demographic groups. Population size estimates were calculated using Microsoft Excel and followed procedures outlined in the WHO/UNAIDS Guidelines on Estimating the Size of Populations Most at Risk to HIV [55].

### Ethical approvals

This research was reviewed and approved by the Institutional Review Boards at the Johns Hopkins Bloomberg School of Public Health (Protocol #00020074) and the collaborating Tribal Nation. This research adhered to the Declaration of Helsinki.

## Results

### Drug use recency and route of administration

In total, *N* = 653 unique surveys were completed. Among the unique surveys, *n* = 80 indicated that the participant had never used any drugs by the routes of administration we asked about and *n* = 72 reported that their substance use histories were limited to marijuana only. After excluding data from these persons, we were left with surveys from *N* = 501 unique persons who had used at least one illicit drug in their lifetime. As shown in Table 1, the degree to which participants had recently engaged in administering drugs by each route of administration were heterogenous. For instance, slightly less than half (48.7%) of participants had never injected drugs, large proportions had injected in the past six months (19.6%) or greater than six months ago (31.7%). There were *N* = 210 persons who reported having used illicit substances by at least one route of administration within the last six months and formed the analytic sample for the remainder of the analyses.

**Table 1:** Recency of drug use by route of administration among people who had used drugs in their lifetime in a County within the jurisdiction of a tribal Nation in the Southern plains of the united States (USA), April-May 2023

	Injected; *N* (%)	Smoked; *N* (%)	Snorted/Sniffed; *N* (%)	Ingested; *N* (%)
**Less than 6 months ago**	98 (19.6)	123 (24.6)	123 (24.6)	114 (22.8)
**Greater than 6 months ago**	159 (31.7)	235 (46.9)	236 (47.1)	243 (48.6)
**Never**	244 (48.7)	143 (28.5)	142 (28.3)	143 (28.6)

*N* = 1 missing data for last drug use by ingestion

### Characteristics of persons who recently used illicit substances (*N* = 210)

Among persons who recently used illicit substances, the mean age was 41.5 years (SD:12.7), and the majority (57.1%) identified as male (Table 2). Most (92.9%) of the sample resided in the focal county. Socio-economic challenges were prevalent, including having not completed high school (33.8%), unemployment (33.8%), homelessness (26.7%), and a recent history of arrest (28.1%). A majority (68.1%) reported having health insurance. Overall, 53.8% of persons who reported recent substance use self-identified at American Indian.

**Table 2:** Characteristics of persons who recently used drugs in a County within the jurisdiction of a tribal Nation in the Southern plains (USA), April-May 2023

	All (*N* = 210); *n* (%)	Self-identify as American Indian (*n* = 113); *n* (%)
**Age (mean**,** SD)**	41.5 (SD: 12.7)	40.3 (SD: 12.5)
**Gender**		
Male	120 (57.1)	67 (59.3)
Female	90 (42.9)	46 (40.7)
**Sexual Orientation**		
Straight	189 (90.0)	100 (88.5)
Sexual Minority	21 (10.0)	13 (11.5)
**Education**		
Less than high school	71 (33.8)	35 (31.0)
High school	95 (45.2)	53 (46.9)
Some college or more	44 (21.0)	25 (22.1)
**Citizen of collaborating Tribal Nation**	88 (41.9)	85 (75.2)
**Hispanic**	17 (8.1)	7 (6.2)
Race*		
White	77 (36.7)	0 (0)
American Indian & White	64 (30.5)	64 (56.6)
American Indian	43 (20.5)	43 (38.1)
American Indian & another race	6 (2.9)	6 (5.3)
Another race/s	20 (9.5)	0 (0)
**Relationship Status**		
Single	99 (47.1)	54 (47.8)
In a relationship	66 (31.4)	43 (38.1)
Married	45 (21.4)	16 (14.2)
**Health insurance**	143 (68.1)	74 (65.5)
**Homeless**	56 (26.7)	36 (31.9)
**Arrest**,** past 6 months**	59 (28.1)	31 (27.4)
**Sex work**,** past 6 months**	13 (6.2)	8 (7.1)
**Went to sleep hungry**, past 6 months**	107 (51.0)	59 (52.2)
**Employment**		
Not working	71 (33.8)	30 (26.5)
Full time	62 (29.5)	39 (34.5)
Part time / odd jobs	77 (36.7)	44 (38.9)

*Participants could select more than one race

**Reported going to sleep hungry once a month or more often

Among persons who self-identified as American Indian, responses to questions about cultural identity were overwhelmingly affirmative. Out of seven questions, the mean number of Indigenous identity and values measures with which participants agreed or strongly agreed was 5.84 (SD: 1.80). The most frequently agreed-with measure was “I think that Indigenous people have a lot to be proud of,” with 92% of participants in agreement. The least frequently agreed-with measure was “I frequently think about the fact that I am Indigenous.”, with 69.0% of participants in agreement (data not shown).

### Recent substance use (*N* = 210)

Among participants who recently injected (Table 3), several types of drugs were used, with methamphetamine (63.3%), prescription opioids (19.4%), and fentanyl (16.3%) being the most frequently reported drugs injected. Among participants who recently smoked drugs, methamphetamine (79.7%), fentanyl (17.9%), and cocaine (9.8%) were the most frequently reported drugs smoked. Among participants who recently snorted or sniffed drugs, methamphetamine (74.8%), prescription pills (20.3%), and cocaine (13.8%) were the most frequently reported drugs snorted or sniffed. Among participants who recently ingested drugs, prescription opioids (37.7%), benzodiazepines (31.6%), and sedatives (22.8%) were the most frequently reported drugs ingested. Use of buprenorphine, suboxone, or methadone was reported by *n* = 19 (9.0%) of people who recently used illicit drugs.

**Table 3:** Substances used by modality of administration among persons who recently used drugs in a County within the jurisdiction of a tribal Nation in the Southern plains (USA), April-May 2023

	Injected (*N* = 98); *n* (%)	Smoked (*N* = 123); *n* (%)	Snorted/Sniffed (*N* = 123); *n* (%)	Ingested (*N* = 114); *n* (%)
Cocaine	6 (6.1)	12 (9.8)	17 (13.8)	
Heroin	13 (13.3)	6 (4.9)	3 (2.4)	
Fentanyl	16 (16.3)	22 (17.9)	6 (4.9)	4 (3.5)
Methamphetamine	62 (63.3)	98 (79.7)	92 (74.8)	
Prescription Opioids	19 (19.4)			43 (37.7)
Benzodiazepines	5 (5.1)			36 (31.6)
Sedatives				26 (22.8)
Prescription stimulants				13 (11.4)
Hallucinogens				9 (7.9)
Prescription pills			25 (20.3)	

### Sexual health, overdose, and harm reduction

Among persons who reported recent illicit substance use, less than half reported past-year testing for sexually transmitted infections (37.3%), HIV (39.2%), and hepatitis C (40.7%) [Table 4]. A significantly greater proportion of persons persons who self-identified as American Indian reported a past year hepatitis C test compared to White participants (p-value: 0.038). Approximately 2.4% reported currently taking pre-exposure prophylaxis (PrEP) for HIV prevention, and 32.4% indicated interest in PrEP. A small proportion of participants (2.9%) indicated they were HIV positive. Nearly one in ten (8.6%) reported having hepatitis C virus infection. Just over one-third (34.0%) of participants had experienced an overdose, with 19.1% having experienced an overdose in the past six months, and 9.0% overdosing more than once during the past six months. Participants also reported having ever witnessed a fatal and nonfatal overdose in their lifetime (24.9% and 45.0%, respectively), and in the past six months (14.8% and 31.6%, respectively).

**Table 4:** Sexual health, overdose, and harm reduction among persons who recently used drugs in a County within the jurisdiction of a tribal Nation in the Southern plains (USA), April-May 2023

	Overall (*N* = 209)*; *n* (%)	Self-identify as American Indian (*N* = 112*); *n* (%)	White (*N* = 77); *n* (%)	*p*-value
**Sexual Health Behaviors**				
STI test within the past year	78 (37.3)	49 (43.8)	25 (32.5)	0.159
HIV test within the past year	82 (39.2)	49 (43.8)	24 (31.2)	0.111
HCV test within the past year	85 (40.7)	53 (47.3)	24 (31.2)	**0.038**
**Overdose Experiences**				
Experienced overdose, ever	71 (34.0)	38 (33.9)	28 (36.4)	0.850
Overdose in past 6 months	40 (19.1)	21 (18.8)	19 (24.7)	0.367
Witnessed a nonfatal overdose, ever	94 (45.0)	52 (46.4)	35 (45.5)	1
Witnessed a nonfatal overdose, past 6 months	66 (31.6)	38 (33.9)	25 (32.5)	0.958
Witnessed a fatal overdose, ever	52 (24.9)	33 (29.5)	16 (20.8)	0.237
Witnessed a fatal overdose, past 6 months	31 (14.8)	19 (17.0)	11 (14.3)	0.689
**Harm Reduction**				
Heard of fentanyl test strips	87 (41.6)	53 (47.3)	31 (40.3)	0.417
Acquired naloxone, past 6 months	50 (23.9)	26 (23.2)	20 (26.0)	0.793
Administered naloxone, past 6 months	42 (20.1)	25 (22.3)	13 (16.9)	0.504
Willing to distribute naloxone	128 (61.2)	69 (61.6)	48 (62.3)	1
Willing to use a public health vending machine	135 (64.6)	68 (60.7)	54 (70.1)	0.240
Willing to distribute take home HIV tests	129 (61.7)	65 (58.0)	51 (66.2)	0.325
Willing to use a needle exchange	113 (54.1)	63 (56.3)	43 (55.8)	1

*There was *N* = 1 person with missing data for sexual health behaviors, overdose, and harm reduction

Approximately four in ten participants (41.6%) had heard of fentanyl test strips. In the past six months, 23.9% of participants reported having gotten naloxone, and 20.1% had administered it to someone experiencing an overdose (Table 4). Willingness to distribute naloxone was reported by 61.2% of the sample. Similarly, a majority of the sample was willing: to use a public health vending machine to access harm reduction supplies such as sterile injection equipment and naloxone (64.6%), to distribute take-home HIV tests to people they knew who used drugs (61.7%), and to use a needle exchange program if it were available (54.1%). With the exception of hepatitis C testing, none of the measures of sexual health, overdose, and harm reduction significantly differed between participants who self-identified as American Indian and White.

### Population estimates among persons who recently used illicit drugs

During the recapture phase, 18 participants indicated they completed the survey during both phases of the study, were not in-phase duplicates, and had recently used at least one illicit drug. Substituting the number of unique persons who had recently used at least one illicit drug during each study phase and the number of participants who participated in both phases (i.e., recaptures) into the equations shown in Appendix A, we estimated that there were 419 [95% CI: 277, 562] persons in the focal county who had recently used illicit drugs. These data translated to an estimated 1.4% population prevalence of recent illicit drug use among residents aged 18 years or older (Table 5). Similarly, we were able to estimate the population sizes of persons who had recently (past six months) smoked drugs and used methamphetamine with resulting prevalences of 0.6% and 0.7%, respectively. Participant counts for each study phase and the number of recaptures are shown in Appendix B.

**Table 5:** Population estimates among persons who recently used drugs in a County within the jurisdiction of a tribal Nation in the Southern plains (USA), April-May 2023

	Population Estimate (95% CI)	Prevalence
Illicit substance use	419 (277, 562)	1.4
Smoked drugs	182 (123, 240)	0.6
Used methamphetamine	198 (134, 262)	0.7

## Discussion

The results of this study demonstrate that the capture-recapture methodology can be applied with community-based participatory methods to successfully estimate the number of persons with recent histories of substance use within American Indian reservation lands. We estimated that 1.4% of the adult population in the focal county had recently used illicit drugs. Notably, the proportions of persons indicating use of opioids and methamphetamines serves as a call to action for local, state, and Federal leaders to enact evidence-based policies and programs aimed at mitigating associated harms. These data parallel trends found throughout the US and may reflect needs for holistic forms of addiction treatment that encompass opioid and stimulant dependence [56, 57]. Given the scale of opioid and stimulant use in our sample and that it spans populations who self-identify as American Indian and other demographic groups, implementing an array of evidence-based programs tailored to local cultures and contexts should be prioritized.

This research demonstrates that people who use drugs want and need harm reduction services, such as access to public health vending machines, syringe services programs, and naloxone. Our data highlight important discrepancies that warrant action. For example, among people who recently used illicit substances, one in five had overdosed in the past six months and one in three had recently witnessed a nonfatal overdose. However, less than one in four persons had acquired naloxone in the past six months. Our data also demonstrate a high degree of altruism and willingness to provide mutual aid among people who use drugs. More than 60% of our sample indicated willingness to distribute naloxone and HIV self-test kits to their peers. Given the rural nature of our study context and limited availability of harm reduction services, future initiatives designed to mitigate substance use harms should invest time and resources in supporting people who use drugs who provide mutual aid to one another.

Coordinated responses across the Tribal, State, and Federal levels may enhance the implementation and overall efficacy of interventions aimed at mitigating substance use harms in American Indian communities. For instance, Tribes could pursue implementing policies that support tailored interventions (e.g., community naloxone distribution) that are anchored in local teachings and values. At the same time, State-level policies should prioritize strengthening partnerships with Tribal Nations and ensuring tribal health facilities, including drug treatment facilities and harm reduction providers, are adequately funded with autonomy to implement programs and policies that reflect Indigenous approaches to health and well-being. At the Federal level, the government could allocate additional funding to Tribal Nations to support scaling up evidence-based harm reduction interventions (e.g., public health vending machines, syringe services programs) to meet underlying population level needs on reservation lands. Taken together, these efforts, informed by population estimation studies, may lead to significant improvements in substance use harms at the population level among American Indian communities.

More than six in ten people who recently used drugs indicated they were willing to use a public health vending machine if one were available. Public health vending machines have been implemented throughout the world and have a robust evidence-base documenting their public health benefits, including increasing access to overdose and infectious disease prevention resources [58–62]. Given that the majority of participants with histories of recent substance use self-identified as American Indian, public health vending machines could be implemented that offer harm reduction supplies (e.g., sterile injection equipment, naloxone) in combination with other resources that align with traditional Indigenous approaches to health and well-being. Although the implementation of public health vending machines on reservation lands is an emerging realm of public health, it is not without precedent. For instance, in 2023, public health vending machines that offered free, anonymous access to sterile injection equipment, naloxone, HIV self-tests, and other public health resources were implemented by the Bois Forte Band of Chippewa in Northern Minnesota with exceptional community support, including unanimous approval from the Reservation Tribal Council [63, 64].

According to the National Academies of Sciences, Engineering, and Medicine, opioid use disorder is “…associated with a 20-fold greater risk of early death due to overdose, infectious diseases, trauma, and suicide.” [65] This finding should be carefully considered in combination with the prevalence of illicit substance. Protecting the lives of people who use drugs may be achieved by increasing access to medications for opioid use disorder (MOUD), such as methadone and suboxone. MOUD utilization carries numerous benefits for people who use drugs, including reductions in opioid use, injection drug use, bloodborne infectious disease transmission risk behaviors, HIV diagnoses, overdose morbidity and mortality, and criminality [65, 66]. In addition, MOUD utilization has been associated with lower healthcare usage and costs than treatment without medications [66]. While the evidence is clear that MOUD save lives and is fiscally beneficial, utilization remains low in rural communities throughout the US [67, 68]. Limited access to MOUD can be attributed to stigma, regulatory requirements, lack of prescriber confidence treating OUD, and inaccurate fears that MOUD medications are “replacing” one drug for another [66, 67, 69, 70]. MOUD utilization may also present tensions with Indigenous communities as some cultural teachings require the absence of psychotropics in order for persons to access traditional healing [71, 72]. Nevertheless, there are many examples of Indigenous communities embracing MOUD in combination with traditional approaches. Initiatives should be launched that provide persons with robust access to MOUD, harm reduction, and traditional approaches to health.

The collaborating Tribal Nation has a large tribally owned health care system with health centers located throughout reservation communities. The health centers provide treatment for OUD such as MOUD, outpatient behavioral health services, and contract with area inpatient facilities for patients requiring more intensive specialized care. The results of the current study may raise some important community health questions about the extent to which the services available for OUD are being accessed and utilized. A recent study examining national data revealed a pattern where access to MOUDs was underutilized by AI/ANs in certain clinic contexts despite this population’s elevated risk [73]. Future work should be conducted to evaluate the degree to which available services align with population-level needs.

This research makes an important contribution to the scientific literature by demonstrating that population estimation methodologies can be successfully integrated with community-based participatory approaches. CBPR is an important methodological orientation in studies conducted in partnership with Indigenous communities given the legacy of unethical and exploitive studies. Throughout this study, researchers at the university and members of the CRC were partners. CRC involvement not only led to important refinements of the survey instrument, but also afforded immediate and wide-scale support for the study at the community level during participant recruitment. The scale and expediency of data collection speaks to the hard work and dedication of the CRC. Further, a majority of our sample identified as American Indian; given the justifiable mistrust many Indigenous Peoples have toward research, our methodological approach demonstrates the utility of anchoring research at the community level. Notably, the volume of data collection afforded several population estimates, including counts of persons who recently used any illicit substance, smoked drugs, and used methamphetamine. These nuances in the population estimates may be used in subsequent efforts to appropriately scale response efforts. This study also adds to emerging evidence that population estimation methodologies can be successfully applied in rural contexts.

As with any population estimation study, there are limitations that should be considered. First, our population estimates are based on participants’ ability to accurately recall their prior participation and self-report it on the survey instrument. This is likely a minor limitation given that persons received memorable incentives, we had extensive study branding, and the CRC was involved in each component of the project. Participants were also instructed that their honesty in reporting prior participation was essential for accurate data collection and that their answers would not prevent them from receiving the incentive. Another limitation is that our data collection efforts focused on areas where people who use drugs were known to congregate. As a result, our data may undercount persons who live in more remote parts of the focal county. It is conceivable that the population of people who use drugs has a degree of transience due to housing instability and/or seasonality. Additionally, we excluded persons from our analyses who did not indicate use of any of the substances we asked about on the survey. These individuals most likely considered alcohol as a form of illicit substance use.Further, this study reflects a single implementation context. There is incredible diversity across hundreds of state and Federally recognized Tribal communities in the US. As a result, our findings may not be translatable. Finally, we were not able to triangulate our findings with local data sources as none comprehensively measured IDU-associated behaviors or outcomes. Despite this limitation, we presented our findings to the collaborating CRC and they felt the population estimates were accurate. We also presented the findings to constituents at the collaborating Tribal Nation who provide services to PWID and they agreed that the data aligned with their understanding of the local population.

In conclusion, this study demonstrates that population estimation methodologies can be integrated with CBPR approaches to quantify the size of populations of people who use drugs on American Indian reservation lands. Our findings provide key insights into the scale of population-level needs for evidence-based harm reduction, MOUD, and traditional Indigenous approaches to health and healing. Reversing trends in substance use morbidity and mortality among persons on reservation lands requires a combination of approaches that are tailored to local cultures and contexts. Future work should be conducted to understand the degree to which population-level needs evolve over time and in response to local initiatives.

## Data Availability

The datasets generated and analyzed during the current study are not publicly available as determined by the data sovereignty agreement with the collaborating Tribal Nation. They are not publicly available due to privacy concerns.

## Appendix A: Population estimation and 95% confidence interval formulas

$$Population{\text{ }}Estimate\,\left( N \right) = \frac{{\left( {C1{\text{ }}X\,C2} \right)}}{M}$$

$$Var\,\left( N \right) = \frac{{\left( {\left( {C1\,X\,C2} \right)\left( {C1\,--\,M} \right)\left( {C2\, - \,M} \right)} \right)}}{{{M^3}}}$$

C1 = Capture Phase Count.

C2 = Recapture Phase Count.

M = Recaptures (Individuals counted in capture and recapture phase).

## Appendix B: Participant counts by study phase

**Table:**

Recent Form of Substance Use	Capture Phase Counts (C1)	Recapture Phase Counts (C2)	Number of Recapture Participants (M)
Illicit substance use	46	164	18
Smoked Drugs	29	94	15
Used methamphetamine	34	93	16

## Abbreviations

CRC: Community Research Council
MOUD: Medications for opioid use disorder
CBPR: Community Based Participatory Research
OUD: Opioid Use Disorder
AI/AN: American Indian/Alaska Native
HIV: Human immunodeficiency virus
US: United States
